# False Troponin Elevation in Pediatric Patients: A Long-Term Biochemical Conundrum Without Cardiac Effects

**DOI:** 10.3390/diagnostics15151847

**Published:** 2025-07-22

**Authors:** Ceren Yapar Gümüş, Taner Kasar, Meltem Boz, Erkut Ozturk

**Affiliations:** 1 Department of Pediatrics, Ordu University Faculty of Medicine, 52200 Altınordu, Ordu, Turkey; 2 Division of Pediatric Cardiology, Department of Pediatrics, Ordu University Faculty of Medicine, 52200 Altınordu, Ordu, Turkey; taner.kasar@hotmail.com; 3 Department of Biochemistry, Ordu Training and Research Hospital, 52200 Altınordu, Ordu, Turkey; meltembozz@gmail.com; 4 Department of Pediatric Cardiology, Basaksehir Cam ve Sakura City Hospital, 34480 Basaksehir, Istanbul, Turkey; erkut_ozturk@yahoo.com

**Keywords:** autoimmune markers, biochemical interference, cardiac biomarkers, false-positive results, macrotroponin, pediatric troponin elevation

## Abstract

**Background/Objectives**: Elevated troponin levels are widely recognized as key biomarkers of myocardial injury and are frequently used in clinical decision making. However, not all instances of troponin elevation indicate true cardiac damage. In some cases, biochemical or immunological interferences may lead to false-positive results. These situations may lead to unnecessary diagnostic interventions and clinical uncertainty, ultimately impacting patient management negatively. This study aims to investigate the underlying mechanisms of false-positive troponin elevation in pediatric patients, focusing on factors such as macrotroponin formation, autoantibodies, and heterophile antibody interference. **Methods**: This retrospective study analyzed data from 13 pediatric patients who presented with elevated cardiac troponin levels between 2017 and 2024. Clinical evaluations included transthoracic echocardiography (TTE), electrocardiography (ECG), coronary computed tomography angiography (CTA), cardiac magnetic resonance imaging (MRI), and rheumatologic testing. Laboratory findings included measurements of cardiac troponins (cTnI and hs-cTnT) and pro-BNP levels. **Results**: Among 70 patients evaluated for elevated troponin levels, 13 (18.6%) were determined to have no identifiable cardiac etiology. The median age of these 13 patients was 13.0 years (range: 9–16), with 53.8% being female. The most common presenting complaints were chest pain (53.8%) and palpitations (30.8%). TTE findings were normal in 61.5% of the patients, and all patients had normal coronary CTA and cardiac MRI findings. Although initial troponin I levels were elevated in all cases, persistent positivity was observed up to 12 months. Median cTnI levels were 1.00 ng/mL (range: 0.33–7.19) at week 1 and 0.731 ng/mL (range: 0.175–4.56) at month 12. PEG precipitation testing identified macrotroponin in three patients (23.1%). No etiological explanation could be identified in 10 cases (76.9%), which were considered idiopathic. All patients had negative results for heterophile antibody and rheumatologic tests. **Conclusions**: When interpreting elevated troponin levels in children, biochemical interferences—especially macrotroponin—should not be overlooked. This study emphasizes the diagnostic uncertainty associated with non-cardiac troponin elevation. To better guide clinical practice and clarify false positivity rates, larger, multicenter prospective studies are needed.

## 1. Introduction

Despite advances in the use of cardiac biomarkers, the interpretation of elevated troponin levels in pediatric patients remains challenging due to the lack of large pediatric-specific studies. While adult cohorts provide well-established reference ranges, pediatric populations often exhibit immune interferences and assay-specific artifacts that complicate diagnostic accuracy. Therefore, additional pediatric-focused investigations are needed to clarify troponin interpretation in children [1,2].

Chest pain is one of the most common reasons for outpatient and emergency department visits in pediatric patients [3,4]. Although cardiac causes represent only a small proportion of these cases, due to physician and parental concerns, troponin testing is often ordered without a comprehensive clinical evaluation. In the pediatric population, the most frequent etiologies of chest pain are musculoskeletal conditions, respiratory tract infections, gastrointestinal problems, or idiopathic causes [5]. Cardiac troponins are highly sensitive and specific biomarkers of myocardial injury. In children, they are particularly important in the diagnosis of cardiac conditions such as myocarditis, which is one of the leading causes of troponin elevation [6]. However, elevated troponin levels in pediatric patients do not always indicate myocardial damage and may represent false-positive results, complicating clinical decision making [7]. Various biochemical interactions—especially the presence of heterophile antibodies, autoantibodies, and macrotroponin complexes—can lead to false-positive troponin elevations [8]. Such cases may result in unnecessary diagnostic procedures and misdirected clinical interventions. One well-documented source of assay interference is the formation of macrotroponin complexes—high molecular weight, immunoglobulin-bound troponin molecules that can persist in circulation despite the absence of myocardial injury. These complexes have been increasingly reported in pediatric case studies and often lead to persistently elevated troponin levels despite normal cardiac imaging and clinical stability, thereby causing diagnostic confusion and unnecessary clinical actions [2,9]. Despite the high sensitivity of troponin assays, they remain vulnerable to biochemical interference, which may cause laboratory results to be misinterpreted outside the appropriate clinical context [10]. The interpretation of troponin levels in pediatric patients differs significantly from adults, and identifying the true cause of elevated values is essential to avoid unnecessary interventions. However, only a limited number of studies have focused on the etiology of elevated troponin levels in children. Most of the existing literature consists of isolated case reports, and large case series are rare [2].

Although troponin testing is widely used in pediatric patients with suspected myocarditis, the prevalence of false-positive troponin elevation remains poorly defined. While adult studies report a false-positive rate ranging from approximately 0.1% to 3.0%—primarily due to analytical interferences such as heterophile antibodies or macrotroponin formation—equivalent pediatric data are lacking [7]. While isolated pediatric case reports have identified macrotroponin interference, structured case series remain rare. Without systematic investigation—including imaging correlation and confirmatory laboratory methods such as polyethylene glycol (PEG) precipitation or immunoglobulin depletion—the true incidence and clinical impact remain unclear [9]. This study addresses this gap by providing a larger series in a pediatric cohort with suspected false-positive troponin elevation [1,2].

Misinterpretation of troponin results in children may lead to unnecessary imaging, referrals, and even hospitalizations. Addressing this diagnostic uncertainty is crucial to optimize healthcare resources and reduce anxiety for both patients and their families.

This study aims to investigate the clinical and laboratory characteristics of pediatric patients with confirmed false-positive troponin elevation and to explore the biochemical mechanisms—particularly macrotroponin formation, autoimmune antibodies, and assay interferences—underlying this phenomenon.

## 2. Materials and Methods

### 2.1. Study Design and Ethical Approval

This study was designed as a retrospective observational analysis of pediatric patients who presented to our hospital between January 2017 and December 2024 and met the predetermined inclusion criteria. The study was conducted by the principles of the Declaration of Helsinki and was approved by the Clinical Research Ethics Committee of Ordu University (Approval No: 26, Date: 2 July 2025).

### 2.2. Inclusion and Exclusion Criteria

#### 2.2.1. Patients Were Included in the Study if They Met the Following Criteria

- Age between 1 month and 18 years;
- Serum cardiac troponin I (cTnI) and/or high-sensitivity cardiac troponin T (hs-cTnT) levels above the upper reference limit (URL), with persistent elevation during follow-up;
- Complete documentation of transthoracic echocardiography (TTE), electrocardiography (ECG), coronary computed tomography (CT) angiography, cardiac magnetic resonance imaging (MRI), and exercise testing;
- Comprehensive laboratory and clinical follow-up data in electronic medical records;
- At least six months of consistent outpatient follow-up.

#### 2.2.2. Exclusion Criteria Were as Follows

- Neonates (under 1 month of age);
- Patients with abnormal findings on coronary CT angiography, cardiac MRI, or exercise testing;
- Patients with follow-up duration shorter than 6 months or incomplete diagnostic data.

### 2.3. Diagnostic Assessment and Laboratory Testing

All included patients underwent the following laboratory and imaging evaluations during admission or the diagnostic process:

- Biochemical Parameters and Complete Blood Count: Cardiac biomarkers (cTnI and hs-cTnT), creatine kinase-MB (CK-MB), pro-BNP, and liver and kidney function tests (Cobas 8000, Roche Diagnostics, Mannheim, Germany).
- Rheumatologic and Autoimmune Testing: Antinuclear antibody (ANA; HELIOS, AESKU GROUP GmbH, Wendelsheim, Germany), rheumatoid factor (RF), anti-double-stranded DNA (anti-dsDNA), anti-Smith antibodies (Sm) (TRITURUS, GRIFOLS, Barcelona, Spain), antistreptolysin O (ASO) titers (Cobas 8000, Roche Diagnostics, Mannheim, Germany), anti-cyclic citrullinated peptide (anti-CCP) antibodies (Atellica Solution, Siemens Healthineers, Erlangen, Germany), erythrocyte sedimentation rate (ESR) (Sistat Diagnosis and Treatment Systems Inc., Ankara, Turkey), C-reactive protein (CRP) (Cobas 8000, Roche Diagnostics, Mannheim, Germany), and complement levels (C3, C4)(BN ProSpec, Siemens Healthineers, Erlangen, Germany).
- Heterophile Antibody Blocking Tube Evaluation: Heterophile antibody blocking tube (HBT) testing (Scantibodies Laboratory Inc., Santee, CA, USA) was performed in patients suspected of false positivity. Before use, the tube was tapped on a hard surface to settle reagents. Then, 500 µL of patient serum was added, mixed gently by inversion, and incubated at room temperature (15–25 °C) for one hour. Troponin levels were reanalyzed using an autoanalyzer.
- Macrotroponin Evaluation: Macrotroponin detection was performed using the polyethylene glycol (PEG) precipitation method. A 25% PEG-6000 solution (Polyethylene Glycol 6000; Merck, Darmstadt, Germany) was mixed with equal volume (250 µL) of patient serum and vortexed (Vortex-Genie 2; Scientific Industries, Bohemia, NY, USA). After 10 min of incubation at room temperature, the mixture was centrifuged (NF 1000; Nüve, Ankara, Turkey) at 4000 rpm for 15 min. Control samples were prepared by mixing 250 µL of normal serum with 250 µL of distilled water. Troponin levels were remeasured from supernatants using the Cobas system [Roche Diagnostics; URL: 0.16 ng/mL for cTnI, 14 ng/L for hs-cTnT]. PEG precipitation percentage and recovery rate were calculated using the following formulas:
- % PEG Precipitated = (Control Troponin − Post-PEG Troponin)/Control Troponin × 100
- % Recovery = 100 − % PEG Precipitated

Cases with recovery rates below 40% were interpreted as macrotroponin positive [1].

- Imaging and Clinical Follow-Up: All patients underwent TTE (Philips Healthcare, Andover, MA, USA), ECG (Nihon Kohden Corporation, Tokyo, Japan), 24-h Holter ECG (SpaceLabs Healthcare, Snoqualmie, WA, USA), coronary CT angiography (Aquilion Lightning; Canon Medical Systems Corporation, Ōtawara, Tochigi, Japan), and cardiac MRI (Optima MR360 1.5T; GE Healthcare, Chicago, IL, USA). Structural and ischemic heart diseases were ruled out based on imaging. Troponin levels, symptoms, and clinical signs were monitored regularly throughout follow-up. Patients with persistent troponin elevation despite normal imaging and clinical findings were diagnosed with “false-positive troponin elevation” [2,10].

### 2.4. Statistical Analysis

Frequencies and percentages were calculated for categorical variables. For numerical variables, descriptive statistics including mean ± standard deviation (SD), median, and minimum–maximum values were reported. Statistical analyses were performed using IBM SPSS software (version 30; IBM Corporation, Armonk, NY, USA).

This study aimed to enhance understanding of the biochemical and clinical aspects of false-positive troponin elevation in pediatric patients and to contribute to evidence-based clinical practice.

## 3. Results

This retrospective analysis reviewed data from 70 pediatric patients presenting with elevated troponin levels between 2017 and 2024. Among them, 13 patients (18.6%) had no identifiable cardiac etiology associated with troponin elevation.

The median age of these 13 patients was 13.0 years (range: 9–16), with 53.8% (*n* = 7) female and 46.2% (*n* = 6) male. Presenting symptoms included chest pain in 53.8% (*n* = 7) and palpitations in 30.8% (*n* = 4). Non-specific symptoms such as abdominal pain, left arm pain, and unilateral hearing loss accompanied by chest pain were observed in individual cases.

TTE findings were normal in 61.5% (*n* = 8). Minor structural findings were detected in 38.5% (*n* = 5), including mild mitral regurgitation (MR) (*n* = 3), mitral valve prolapse with MR (*n* = 1), and a small secundum atrial septal defect (ASD) with septal aneurysm (*n* = 1). ECG findings were normal in five patients (38.5%). The remaining patients exhibited ST–T wave changes (*n* = 5), supraventricular extrasystole (SVES) (*n* = 1), sinus tachycardia (*n* = 1), and ventricular extrasystole (VES) (*n* = 1). All patients had normal coronary CT angiography and cardiac MRI. One patient also underwent conventional coronary angiography, which was normal.

Between 2017 and 2024, a total of 70 patients were followed in our clinic with a preliminary diagnosis of myocarditis. Myocarditis was diagnosed based on the 2025 ESC Working Group on Myocardial and Pericardial Diseases criteria, incorporating clinical symptoms and imaging findings [11]. Among these, 13 patients were ultimately diagnosed with false-positive troponin elevation due to persistently elevated cardiac troponin levels, absence of pathological findings on imaging, and normal proBNP levels. All 13 patients tested negative for rheumatologic and heterophile antibody panels. Demographic and clinical characteristics are presented in Table 1.

**Figure 1:**
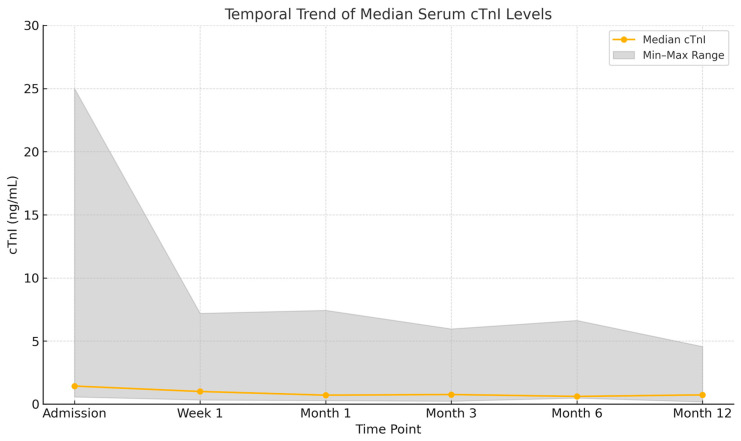
Temporal trend of median serum cTnI levels over a 12-month follow-up. The shaded area represents the minimum and maximum values observed at each interval.

Although cTnI values gradually declined during follow-up, persistently elevated levels were observed up to 12 months. Median cTnI levels at each time point are summarized in Table 2 and visualized in Figure 1.

To identify potential biochemical causes of the elevated troponin levels, macrotroponin testing was performed in all patients using PEG precipitation. Macrotroponin was detected in three patients (23.1%): one tested positive for hs-cTnT, and two for cTnI. In the remaining 10 patients (76.9%), no specific etiology could be identified, and these cases were classified as idiopathic false-positive troponin elevation. The discordance between biochemical findings and imaging/laboratory results supported the diagnosis.

Hs-cTnT levels were measured in selected patients and found to be within the normal range in the majority. One patient showed an elevated hs-cTnT value. However, their proBNP level was normal, and no structural or functional cardiac pathology was observed on imaging. These findings are detailed in Table 2.

Regarding treatment, 12 patients (92.3%) received anti-inflammatory monotherapy, while one patient (7.7%) was treated with a combination of anti-inflammatory therapy and propranolol. During follow-up, no arrhythmias, newly emerging symptoms, or cardiac dysfunction were observed in any patient.

## 4. Discussion

In this study, false-positive troponin elevation was identified in 18.6% of 70 pediatric patients initially evaluated for elevated troponin levels. This notable proportion highlights the potential role of biochemical interferences in generating falsely elevated cardiac biomarkers. A review of the literature reveals that no clear data exist on the incidence of false-positive troponin elevation among patients followed with a preliminary diagnosis of myocarditis. The mismatch between elevated troponin levels and normal clinical or imaging findings underscores the importance of considering false positivity in the differential diagnosis.

Our study stands out as one of the few pediatric case series with a relatively large sample size addressing this issue, whereas existing publications are typically limited to isolated case reports. Among the 70 patients presenting with elevated troponin, 57 (81.4%) were ultimately diagnosed with myopericarditis or perimyocarditis based on the American College of Cardiology (ACC) guidelines. In the remaining 13 cases (18.6%), however, a definitive diagnosis of myocarditis could not be established, and these patients were identified as having false-positive troponin elevation [11]. Of the 70 patients with elevated troponin, only 13 had persistent troponin elevation without any clinical, imaging, or laboratory evidence of myocardial injury and were therefore classified as having false-positive results. While troponin levels returned to normal within a few weeks in most myocarditis-confirmed patients (*n* = 57), persistent elevation was documented exclusively in the 13 false-positive cases throughout the 12-month follow-up. According to the literature, elevated cTnI levels in children are most commonly associated with cardiac conditions such as myopericarditis and perimyocarditis [12,13,14].

These diagnoses were supported by patients’ medical history, physical examination findings, laboratory data, ECG, and TTE results. While troponin levels are generally expected to return to normal within 2–3 weeks following myocarditis, persistent elevation was observed in the patients included in our study [14]. In addition, cardiac MRI performed in these patients revealed no findings suggestive of myocarditis, and all patients had pro-BNP levels within the normal range. Cardiac troponins are the most specific biochemical markers of myocardial injury and play a critical role in the diagnosis of acute coronary syndromes in the adult population. However, in children, elevated troponin levels are not always associated with underlying cardiac disease and may result from various forms of biochemical interference, leading to false-positive results [7,14]. This underscores the importance of a cautious and comprehensive approach in clinical decision making.

One of the most frequently reported causes of false-positive troponin elevation is the presence of macrotroponin complexes. These are formed when troponin molecules bind to circulating immunoglobulins, thereby prolonging their half-life in the bloodstream. This complex formation can result in artificially high troponin values despite the absence of myocardial injury [15]. In most cases, cardiac function remains unaffected. In addition to macrotroponin, heterophile antibodies, autoantibodies, and analytical issues within troponin immunoassays may also contribute to false-positive results [7]. Warner and Marshall reported a 5% incidence of macrotroponin I in patients undergoing high-sensitivity cTnI testing [16]. Circulating autoantibodies against cTnI have been observed in up to 12.7% of healthy individuals [17]. Heterophile antibodies can lead to nonspecific interactions in immunoassays, which may be verified using serial dilutions or heterophile blocking tubes [18]; however, such methods may not be available in every laboratory.

In our study, macrotroponin positivity was found in only 3 of 13 patients with false troponin elevation. In the remaining 10 patients, no positivity associated with known interferences, presence of autoantibodies, heterophil antibodies, and rheumatologic tests was found. This suggests that troponin elevation is largely associated with idiopathic causes. In the literature, it is reported that false troponin elevation is most commonly associated with macrotroponin positivity [10,16,17,19]. Adamczyk et al. reported that approximately 70–80% of false troponin elevations in pediatric patient groups were caused by macrotroponin or heterophile antibodies [17]. However, in our study, only 23.1% showed macrotroponin positivity, and the cause of the remaining 76.9% of false troponin elevation could not be explained. This makes it necessary to emphasize the concept of “idiopathic false troponin elevation,” which is rarely reported in the literature but has great clinical importance. In clinical practice, macrotroponin-induced false troponin elevation is usually associated with cardiac cTnI and is less commonly reported with cTnT [20]. However, in our study, the association of persistence of hs-cTnT levels with macrotroponin positivity in one patient is an important contribution to the literature in terms of showing that cTnT may also be affected in this phenomenon.

The reliability of troponin tests may vary depending on the analytical methods used. Hs-cTnT tests are known to reduce false positive rates [21]. High-sensitivity troponin assays have been shown to improve diagnostic accuracy in adult populations, but their specificity in pediatric cohorts remains uncertain [21].

However, this test may not be available in all centers. Additionally, a recent study reported that long-term false-positive hs-cTnI results can occur due to macrotroponin complex formation, which highlights the variability across assay platforms [19]. The fact that patients with elevated hs-cTnT had normal proBNP levels and no significant findings on advanced cardiac evaluation further suggests that hs-cTnT alone may not be sufficient for the differential diagnosis of false troponin elevation.

These observations highlight the need to interpret troponin levels in conjunction with clinical context and complementary diagnostic tools. When clinical suspicion exists, confirmation with an alternative assay may be essential. Wider implementation of advanced biochemical tests such as hs-cTnT could help reduce unnecessary procedures and improve patient management.

The data presented in this study support the necessity of evaluating troponin results alongside clinical findings and imaging, particularly in pediatric patients. When inconsistencies are noted between troponin levels and clinical presentation, the possibility of false positivity should be seriously considered to avoid unnecessary treatments and interventions.

Understanding the underlying causes of false-positive results is crucial in minimizing invasive procedures and reducing patient and parental anxiety. In cases of persistent troponin elevation, macrotroponin complexes should be specifically investigated. Clinicians should be aware that isolated laboratory results should not guide management in the absence of corroborative clinical evidence. The clinical scenarios in which false-positive troponin elevation should be suspected are summarized in Table 3, along with supporting references from the literature. Further large-scale, prospective studies are needed to clarify the prevalence and underlying mechanisms of macrotroponin-related and idiopathic false troponin elevations in the pediatric population.

Based on the findings of this study and the previously reported literature, clinicians may consider the possibility of false-positive troponin elevation in the following scenarios. However, these observations should be interpreted cautiously given the small sample size and descriptive nature of this study:

- When there is a mismatch between clinical presentation and troponin levels [7,14];
- When imaging findings are unremarkable [7,11,13];
- When cardiac biomarkers such as proBNP are within normal limits [19,21];
- When troponin levels remain elevated for an unexpectedly long period [7,10,16];
- When ECG and TTE findings are normal (no direct ref., but supported by study cohort findings);
- When there is no history of underlying cardiac disease or risk factors (data derived from the current study);
- When macrotroponin is confirmed by PEG precipitation [2,10,16];
- When autoantibody and heterophile antibody tests are negative [7,17,18];
- When significant differences are observed between test methods [21];
- When laboratory values do not respond to treatment (data derived from the current study).

These are summarized in Table 3.

### Limitations

Although this study provides valuable insight into false-positive troponin elevation in pediatric patients, several limitations should be noted. First, the relatively small sample size (*n* = 13) limits the generalizability of the findings to broader pediatric populations. Second, the accuracy and consistency of clinical and laboratory assessments were dependent on the initial diagnostic procedures, introducing the possibility of bias due to the retrospective design. Additionally, comprehensive data on genetic predispositions or other biochemical interferences potentially contributing to false-positive troponin results were not available. Another limitation is that the study was conducted at a single institution, which may not fully reflect the heterogeneity of pediatric cases seen in different healthcare settings. Finally, the 12-month follow-up period may not have been sufficient to capture long-term trends or recurrence patterns in troponin levels.

Moreover, the small sample size and retrospective design without inferential statistical analysis limit the generalizability of our findings. Thus, the proposed clinical scenarios should be viewed as exploratory rather than definitive.

## 5. Conclusions

This study highlights the potential role of macrotroponin complexes, autoantibodies, and heterophile antibodies in the differential diagnosis of false-positive troponin elevation in children. Our findings suggest that troponin levels may remain persistently elevated in the absence of cardiac involvement and may, in some cases, be attributable to idiopathic causes. To prevent unnecessary diagnoses, treatments, and costly interventions, laboratory results should always be interpreted in conjunction with clinical findings. Increasing awareness of this issue among clinicians may lead to more cautious and evidence-based management strategies in pediatric practice. Further multicenter studies are needed to validate these findings and better characterize false-positive troponin elevations in pediatric populations.

## Figures and Tables

**Table 1:** Baseline characteristics of the patients; findings from TTE, ECG, CT, and MRI; rheumatologic test results; heterophile antibody status; proBNP levels; and treatment modalities.

		n (%)
Gender	Male	6 (46.2)
Age	Median (Min-Max)	13.0 (9–16)
Presenting Symptoms	Palpitations	4 (30.8)
	Chest pain	6 (46.2)
	Abdominal pain	1 (7.7)
	Unilateral hearing loss, epistaxis, and chest pain	1 (7.7)
	Left arm pain	1 (7.7)
TTE Findings	Small secundum ASD, atrial septal aneurysm, mild MR	1 (7.7)
	Mild MR	3 (23.1)
	MVP, MR	1 (7.7)
	Normal	8 (61.5)
ECG Findings	Normal	5 (38.5)
	Sinus tachycardia	1 (7.7)
	ST–T wave changes	5 (38.5)
	SVES	1 (7.7)
	VES	1 (7.7)
Coronary CT Angiography	Normal	12 (92.3)
Conventional Coronary Angiography	Normal	1 (7.7)
Cardiac MRI	Normal	13 (100)
Rheumatologic Test Results	Negative	13 (100)
Heterophile Antibody Test	Negative	13 (100)
Treatment	Anti-inflammatory therapy	12 (92.3)
	Propranolol and anti-inflammatory therapy	1 (7.7)
Total		13 (100)

ASD: atrial septal defect; MR: mitral regurgitation; MVP: mitral valve prolapse; SVES: supraventricular extrasystole; VES: ventricular extrasystole; TTE: transthoracic echocardiography; ECG: electrocardiography; MRI: magnetic resonance imaging.

**Table 2:** Serial measurements of cTnI levels (ng/mL) and macrotroponin status of the patients.

	Median	Min-Max
Admission	1.43	0.57–25.00
Week 1	1.00	0.33–7.19
Month 1	0.71	0.28–7.43
Month 3	0.76	0.23–5.96
Month 6	0.61	0.48–6.63
Month 12	0.73	0.18–4.56
Hs-cTnT Levels (pg/mL)		n (%)
	40.5	1 (7.7)
	Normal	12 (92.3)
Macrotroponin Status	Negative	10 (76.9)
	Positive	3 (23.1)
Total		13 (100)

cTnI: cardiac troponin I; hs-cTnT: high-sensitivity cardiac troponin T.

**Table 3:** Summary of clinical scenarios in which false-positive troponin elevation should be considered in pediatric patients.

Clinical Scenario	Supporting References
Mismatch between clinical presentation and troponin levels	[5,13]
Unremarkable cardiac imaging findings (TTE, MRI, CT angiography)	[5,10,12]
Normal cardiac biomarkers such as proBNP	[17,19]
Troponin levels remain elevated over a prolonged period (e.g., 12 months)	[5,7,14]
Normal ECG and TTE findings	[5]
No history of underlying cardiac disease or identifiable risk factors	[2,4]
Macrotroponin positivity confirmed by PEG precipitation	[7,8,14]
Negative autoantibody and heterophile antibody test results	[5,15,16]
Significant differences between troponin assay platforms (e.g., hs-cTnI vs. hs-cTnT)	[17,18]
Laboratory values do not respond to standard treatments (e.g., anti-inflammatory medications)	Data derived from the present study

## Data Availability

Patient data can be shared if requested.

## Abbreviations

The following abbreviations are used in this manuscript:

ACC	American College of Cardiology
ANA	Antinuclear Antibody
ASO	Antistreptolysin O
cTnI	Cardiac Troponin I
cTnT	Cardiac Troponin T
CK-MB	Creatine Kinase-MB
CRP	C-Reactive Protein
CT	Computed Tomography
ECG	Electrocardiography
ESR	Erythrocyte Sedimentation Rate
HBT	Heterophile Blocking Tube
hs-cTnT	High-Sensitivity Cardiac Troponin T
MRI	Magnetic Resonance Imaging
MR	Mitral Regurgitation
MVP	Mitral Valve Prolapse
PEG	Polyethylene Glycol
RF	Rheumatoid Factor
SVES	Supraventricular Extrasystole
TTE	Transthoracic Echocardiography
URL	Upper Reference Limit
VES	Ventricular Extrasystole

## References

[1] Lam L., Ha L., Gladding P., Tse R., Kyle C. (2021). Effect of macrotroponin on the utility of cardiac troponin I as a prognostic biomarker for long term total and cardiovascular disease mortality. Pathology.

[2] Harberg M.R., Al-Mousily M.F., Akter T., Babic N., Jackson L.B. (2023). Persistent elevation of troponin I in a pediatric patient resulting from macrotroponin complex. Pediatrics.

[3] Fogliazza F., Cifaldi M., Antoniol G., Canducci N., Esposito S. (2024). Approaches to pediatric chest pain: A narrative review. J. Clin. Med..

[4] Alsabri M., Elshanbary A.A., Nourelden A.Z., Fathallah A.H., Zaazouee M.S., Pincay J., Nakadar Z., Wasem M., Aeder L. (2024). Chest pain in pediatric patients in the emergency department—Presentation, risk factors and outcomes—A systematic review and meta-analysis. PLoS ONE.

[5] Saleeb S.F., Li W.Y.V., Warren S.Z., Lock J.E. (2011). Effectiveness of screening for life-threatening chest pain in children. Pediatrics.

[6] Kanaan U.B., Chiang V.W. (2004). Cardiac troponins in pediatrics. Pediatr. Emerg. Care.

[7] Chaulin A.M. (2022). False-positive causes in serum cardiac troponin levels. J. Clin. Med. Res..

[8] Michielsen E.C.H.J., Bisschops P.G.T., Janssen M.J.W. (2011). False positive troponin result caused by a true macrotroponin. Clin. Chem. Lab. Med..

[9] Lucas R., Roberts P. (2020). Macrotroponin as a cause for an elevated troponin in a 14-year-old boy. J. Paediatr. Child Health.

[10] Laguë M., Turgeon P.Y., Thériault S., Steinberg C. (2022). A false-positive troponin assay leading to the misdiagnosis of myopericarditis. CMAJ.

[11] Drazner M.H., Bozkurt B., Cooper L.T., Aggarwal N.R., Basso C., Bhave N.M., Caforio A.L.P., Ferreira V.M., Heidecker B., Writing Committee (2025). 2024 ACC expert consensus decision pathway on strategies and criteria for the diagnosis and management of myocarditis. J. Am. Coll. Cardiol..

[12] Brown J.L., Hirsh D.A., Mahle W.T. (2012). Use of troponin as a screen for chest pain in the pediatric emergency department. Pediatr. Cardiol..

[13] Dionne A., Kheir J.N., Sleeper L.A., Esch J.J., Breitbart R.E. (2020). Value of troponin testing for detection of heart disease in previously healthy children. J. Am. Heart Assoc..

[14] Yoldaş T., Örün U.A. (2019). What is the significance of elevated troponin I in children and adolescents? A diagnostic approach. Pediatr. Cardiol..

[15] Salaun E., Drory S., Coté M.-A., Tremblay V., Bédard E., Steinberg C., Paré D., Kim O., Cieza T., Coté N. (2024). Role of antitroponin antibodies and macrotroponin in the clinical interpretation of cardiac troponin. J. Am. Heart Assoc..

[16] Warner J.V., Marshall G.A. (2016). High incidence of macrotroponin I with a high-sensitivity troponin I assay. Clin. Chem. Lab. Med..

[17] Adamczyk M., Brashear R.J., Mattingly P.G. (2009). Circulating cardiac troponin-I autoantibodies in human plasma and serum. Ann. N. Y. Acad. Sci..

[18] Lakusic N., Sopek Merkas I., Lucinger D., Mahovic D. (2021). Heterophile antibodies, false-positive troponin, and acute coronary syndrome: A case report indicating a pitfall in clinical practice. Eur. Heart J. Case Rep..

[19] Sušić T., Miler M., Nikolac Gabaj N., Tešija Kuna A., Kordić K., Ilić V., Vinter O. (2024). Long-term false positive hsTnI on Alinity I probably caused by macrotroponin complex: Case report. Clin. Biochem..

[20] Akhtar Z., Dargan J., Gaze D., Firoozi S., Collinson P., Shanmugam N. (2020). False-positive troponin elevation due to an immunoglobulin G cardiac troponin T complex: A case report. Eur. Heart J. Case Rep..

[21] Krychtiuk K.A., Newby L.K. (2024). High sensitivity cardiac troponin assays: Ready for prime time!. Annu. Rev. Med..

